# Thymic Tumor Extension into the Heart, a Rare Finding Found by Point-of-Care Ultrasound

**DOI:** 10.7759/cureus.724

**Published:** 2016-08-04

**Authors:** Elizabeth Kaufman, Michelle Hunter-Behrend, Eric Leroux, Laleh Gharahbaghian, Viveta Lobo

**Affiliations:** 1 Department of Emergency Medicine, Stanford University School of Medicine

**Keywords:** trans-thoracic echocardiography, tumors, thymic tumor, cardiac neoplastic tumors, emergency, point-of-care diagnostics, ultrasound, chest radiology, emergency department

## Abstract

We report a cardiac mass detected by point-of-care ultrasound performed within the emergency department on a 65-year-old male with thymic cancer who presented with chronic cough and fever. Results from the initial emergency workup, which included blood tests, urinalysis, and a computerized tomography with angiography scan with venous phasing of the chest, did not result in a definitive diagnosis. A point-of-care echocardiogram was performed to evaluate for possible infective endocarditis, but alternatively identified a large mass in the right atria and ventricle. The mass was later confirmed to be metastatic tumor from the patient’s known thymic cancer. This case emphasizes the vital role ultrasound can play in the acute care setting.

## Introduction

Cardiac neoplastic tumors are uncommon findings categorized as primary benign tumors, primary malignant tumors, or metastatic tumors [1]. Trans-thoracic echocardiography remains the first-line imaging modality for diagnosis and assessment of these tumors because of its widespread availability, lack of radiation exposure, and versatility [2-3]. We present a case where point-of-care transthoracic echocardiography identified a cardiac mass in a patient presenting with fever and cough, dramatically altering the patient’s hospital course and management.

## Case presentation

A 65-year-old male with a newly diagnosed mediastinal thymic mass and pulmonary embolism presented to the emergency department (ED) with a reported fever of 103°F and chronic cough that had started at home seven hours prior to arrival. He complained of generalized fatigue without chest pain, abdominal pain, or nausea. The patient had started enoxaparin four days prior to presentation. The remainder of his review of systems was negative. His initial vital signs showed a pulse rate of 123 beats/min, respiratory rate of 20 breaths/min, blood pressure of 129/72 mm Hg, oxygen saturation of 95% on room air, and temperature of 38.7°C (101.6°F). He was alert, oriented, and answered all questions appropriately. His cardiopulmonary exam revealed normal heart and lung sounds. The remainder of his physical exam was unremarkable. The patient consented to the treatment.

An intravenous line was placed, and the patient received two liters of normal saline and acetaminophen by mouth for his fever. The working differential diagnosis included pneumonia, pulmonary abscess or empyema, infective endocarditis, new pulmonary emboli, or new tumor burden. An evaluation was initiated that included laboratory studies, urinalysis, and radiographic studies. Laboratory results were significant for a total white blood cell (WBC) count of 18 x 10^3^/μL (ref. 4-11 x 10^3^/μL) with a corresponding neutrophilic predominance. A computerized tomography angiography (CTA) scan with venous phasing of his chest was interpreted by radiology as unchanged pulmonary emboli and anterior mediastinal mass along with a small, new right pleural effusion.

**Figure 1:**
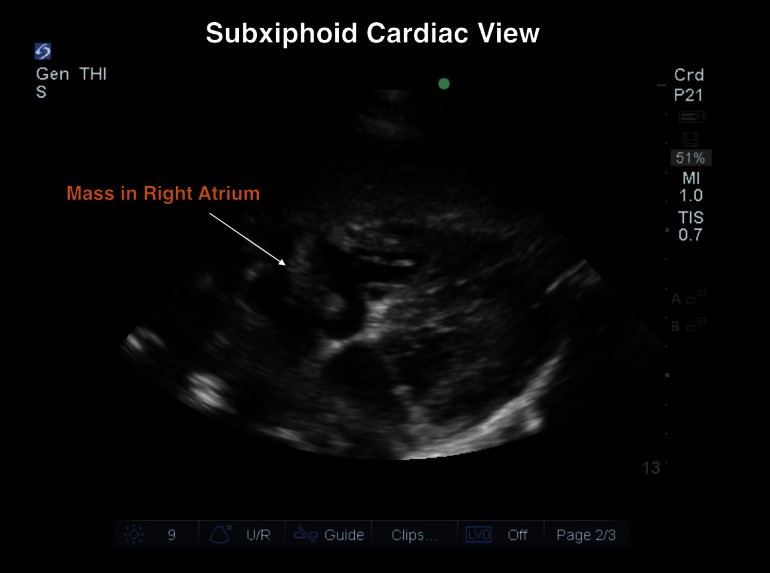
Subxiphoid Cardiac View: Mass in Right Atrium

**Figure 2:**
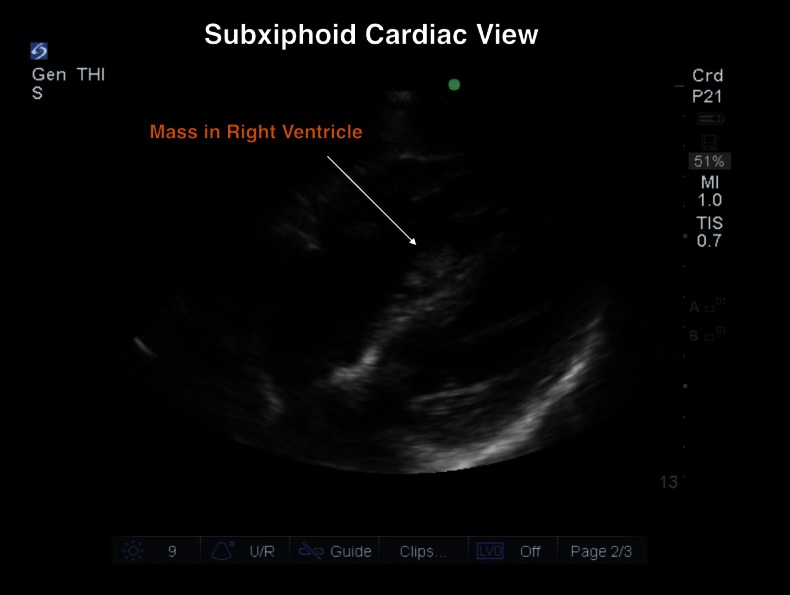
Subxiphoid Cardiac View: Mass in Right Ventricle

Given the exclusion of an obvious infectious source from the CTA chest scan, a point-of-care ultrasound was performed using a 5-1 MHz probe (Sonosite, Bothell, WA) to further evaluate the heart for signs of infective endocarditis. The examination revealed a large mobile mass in the right atrium (RA) and right ventricle (RV), concerning for either new tumor burden or thrombus (Figures 1-2, Video 1). Given the mobility of the mass and the absence of valvular vegetations, infective endocarditis was less likely. However, due to the fever, the patient was given broad-spectrum antibiotics intravenously. Following treatment in the ED, reassessment of the patient revealed improved vitals signs with a pulse rate of 105 beats/min, respiratory rate of 18 breaths/min, blood pressure of 130/75 mm Hg, and a temperature of 37.6°C (99.6°F). The oncology medical service was consulted for hospital admission.

**Video 1:** Apical 4-Chamber: Mass in Right Heart

This patient's hospital course was significant for increasing right pleural effusion which required a thoracentesis. A transthoracic echo by the cardiology service was performed, reaffirming the RA/RV mobile mass, now considered to be tumor extension from the mediastinum with associated thrombus not visualized on prior CTA imaging. The patient's blood culture results remained negative during his hospital course, and he developed no other signs of infection. His original fever was thought to be part of his increased tumor burden. After a seven-day course of intravenous antibiotics, the patient improved clinically and remained afebrile, and his WBC count returned to within normal range. The oncology service adjusted his cancer treatment plan given this new finding. Antibiotics were discontinued, and the patient was discharged home in stable condition after nine days.

## Discussion

Point-of-care ultrasonography has been shown to assist in the rapid diagnosis and management of patients in the ED [4]. Despite each application of point-of-care ultrasound having a focused assessment profile, it is not uncommon to incidentally diagnose or visualize pathology that falls outside its intended focus. In the case presented, an ultrasound intended to evaluate for infective endocarditis instead identified a large mass in the right side of the heart; the mass was later confirmed to be a metastatic tumor from the patient’s known thymus cancer. Given that this mass was not seen on the chest CTA performed during the same visit, the finding may have been missed if the point-of-care ultrasound was not performed in the ED.

Metastatic tumors make up the large majority of cardiac tumors and are most often metastases from lung (35-40%), breast (10%), or hematologic cancers (10-20%). Less frequently, metastases originate from renal, hepatic, adrenal, and thyroid tumors [5-6]. While thymic tumors are rare slow growing tumors, they are the most common primary tumor of the mediastinum accounting for 40% of all primary mediastinal masses. However, due to the overall rarity of thymic tumors, it is difficult to recognize metastatic tumors within the heart as thymic in origin and even more challenging to diagnose thymic tumors that metastasize outside the thorax cavity. Extra-thoracic thymic metastases occur only 3-6% of the time [7]. Patients with cardiac tumors commonly present with symptoms including chest pain, cough, syncope/dyspnea [8]. Extra-cardiac manifestations such as abdominal pain can also occur, as discussed in a case reported by Pourmand et al [9].

## Conclusions

The use of bedside ultrasound in the ED is expanding exponentially with the addition of many new applications and indications for use. With the rise of advanced echocardiography, cardiac pathology may be diagnosed earlier in the ED, resulting in prompt evaluation and treatment. In this case report, our patient had a common presentation, fever, and cough. A corresponding chest CTA did not demonstrate a cause of his symptoms or the incidental intracardiac mass which was successfully identified by point-of-care ultrasound. Early identification of the metastasis via ED ultrasonography ensured the correct management of this patient's cancer.
